# The Potential Roles of Exosomal Non-Coding RNAs in Hepatocellular Carcinoma

**DOI:** 10.3389/fonc.2022.790916

**Published:** 2022-02-24

**Authors:** Wei Wang, Li-Ping Hao, Haizhu Song, Xiao-Yuan Chu, Rui Wang

**Affiliations:** ^1^Department of Medical Oncology, Jinling Hospital, Nanjing Medical University, Nanjing, China; ^2^Department of Medical Oncology, School of Medicine, Jinling Hospital, Nanjing University, Nanjing, China

**Keywords:** exosomal non-coding RNAs, hepatocellular carcinoma, exosome, extracellular vesicles, diagnostic biomarkers, therapeutic target

## Abstract

Hepatocellular carcinoma (HCC) is the sixth highest-incidence cancer and the 4th most deadly cancer all over the world, with a high fatality and low diagnostic rate. Nowadays, Excessive alcohol consumption, type-2 diabetes, smoking and obesity have become some primary risk factors of HCC. As intercellular messenger transporting information cargoes between cells, exosomes are a type of extracellular vesicles (EVs) released by most types of cells including tumor cells and non-tumor cells and play a pivotal role in establishing an HCC microenvironment. Exosomes, and more generally EVs, contain different molecules, including messenger RNAs (mRNAs), non-coding RNAs (ncRNAs), proteins, lipids and transcription factors. The three main ncRNAs in exosomes are microRNAs (miRNAs), long non-coding RNAs (lncRNAs), circular RNAs (circRNAs). NcRNAs, identified as essential components, are selectively sorted into exosomes and exosomal ncRNAs show great potential in regulating tumor development, including proliferation, invasion, angiogenesis, metastasis, immune escape and drug resistance. Here, we chiefly review the formation and uptake of exosomes, classification of exosomal ncRNAs and current research on the roles of exosomal ncRNAs in HCC progression. We also explored their clinical applications as new diagnostic biomarkers and therapeutic avenues in HCC.

## Introduction

As a major adult liver malignancy, Hepatocellular carcinoma (HCC) accounts for 75-85% of all primary liver cancers with easy metastasis, poor prognosis and low survival rate (1). Hepatitis B virus (HBV) infection is the main reason of liver cancer in China, while hepatitis C virus (HCV) infection is more frequent in Western countries (2). Other etiologies including type-2 diabetes, smoking and obesity, alcoholic liver disease, non-alcoholic fatty liver disease (NAFLD), non-alcoholic steatohepatitis (NASH) can result in HCC (3–5). HCC occurs largely on the basis of liver cirrhosis (LC) (4, 6, 7). In the past few years, accumulating evidence has demonstrated the regulatory roles of diverse categories of non-coding RNAs (ncRNAs) in liver carcinogenesis associated with many etiologies, including HBV, HCV and NAFLD. For instance, HCV infection could suppress miR-122 and miR-122 targets to induce liver carcinogenesis. Dysregulated expression of miRNA has been connected with NAFLD-associated HCC development (8). In China, 5-year survival rates of HCC have been reported to be as low as 12% (9), mainly ascribed to its insidious onset, lack of obvious symptoms in the early stage, rapid progression and difficulty of early effective diagnosis through current non-invasive clinical screening technology like serum alpha-fetoprotein (AFP) and imaging examination (10). As a result, when diagnosed, most patients with HCC are at an advanced stage. They usually accompany with multiple intrahepatic or distant metastasis and miss the opportunity of radical treatments such as surgical resection or orthotopic liver transplantation. Therapeutic strategies for liver cancer include radical surgical resection, radiofrequency ablation (RFA), systemic chemotherapy, liver transplantation and transcatheter arterial chemoembolization (TACE). However, due to poor liver function, current treatment choices for advanced HCC are limited to palliative treatments or strive for second-stage surgery through chemotherapy, interventional therapy and targeted therapy (11). Sorafenib was first identified as an oral multitargeted tyrosine kinase inhibitor drug for systemic chemotherapy in advanced HCC patients with metastasis and can prominently extend the median survival of patients. However, drug resistance gradually emerges during the later stage of treatment, which become a major difficulty in the treatment of liver cancer (12). Therefore, it is an urgent need to improve the early diagnostic rate and explore effective treatment for HCC.

EVs can be divided into two main types: exosomes and ectosomes or microvesicles, on the basis of their differential modes of biogenesis and size (13, 14). Exosomes correspond to a series of nano-extracellular vesicles with a phospholipid bilayer membrane (15, 16) with 30–100 nm in diameter and 1.13–1.19 g/mL in density (16–19). Microvesicles or ectosomes, in comparison to exosomes, range in size from exosome-like EVs of 50nm to EVs as large as 10μm in diameter (13). Observed by transmission electron microscopy (TEM), exosomes present distinctive cup-shaped or disc-shaped morphology (20). Exosomes are distributed in the majority of body fluids, including blood, urine, breast milk, saliva, tears, cerebrospinal fluid, bronchial lavage fluid, amniotic fluid and synovial fluid (21–23). These nanostructures usually contain proteins, lipid molecules, nucleic acids and other inorganic substances such as Ca2+ (16, 20). Nucleic acids in exosomes mainly comprise messenger RNAs (mRNAs), long noncoding RNAs (lncRNAs), ribosomal RNAs (rRNAs), microRNAs (miRNAs/miRs), transfer RNAs (tRNAs), circular RNAs (circRNAs), cell free DNAs (cfDNAs) and mitochondrial DNAs (mtDNAs) (24). Many types of cells may induce the release of exosomes such as tumor cells, endothelial cells, mesenchymal cells, B lymphocytes, T cells, dendritic cells and mast cells (25). In addition, extensive evidence have confirmed that exosomes might play a central part in the initiation, development, diagnosis and therapy of liver cancer (26). Furthermore, the biologically active contents of exosomes have provided novel approaches for the establishment of efficient clinical diagnostics and therapeutic strategies (27). Despite several breakthroughs have been made in exploring the exosomes, further studies are required to fully elucidate the specific biological role of exosomes.

NcRNAs are a series of RNAs unable to encode protein which are divided into lncRNAs (>200 nucleotides) and miRNAs (19–25 nucleotides) according to their length. NcRNAs regulate gene expression post-transcriptionally by translational inhibition or degradation of target mRNA (28, 29). A growing body of studies have demonstrated that exosomal ncRNAs are involved in the potential regulation of tumor metastasis, angiogenesis, immune escape, metabolic reprogramming and drug resistance. Exosomal ncRNAs become endogenous tumor regulators in the body (30), and their dysregulation directly associated with the onset and progression of carcinogenesis (31). More importantly, ncRNAs inside the exosome are stable because the double-layer lipid membrane of exosomes can protect ncRNAs from the ribonuclease-mediated degradation. Thus, the integrity and function of the RNAs are not changed (32). It has been found that there is an obvious ncRNA change in HCC patients’ exosomes that is associated with HCC development, angiogenesis and metastasis in previous studies of ncRNAs (25). This review, we aim to address the biology of exosomes, how exosomal ncRNAs contribute to the development of HCC with a major focus on their potential clinical applications in diagnosis and therapy.

## The Structure and Biogenesis of Exosomes

EVs were firstly discovered in mature sheep reticulocytes by Johnstone and coworkers in 1983 and named them as exosomes. They were originally thought of as “fragments” of cells (33). In 1996, It has been confirmed by Raposo and coworkers that exosomes play a crucial role in B cell antigen presentation and cause T cell responses (34). Therefore, exosomes have seen a progressive increase in popularity in recent years and the imperative role of exosomes in tumor diagnosis and treatment has gradually attracted people’s attention. However, the term “exosomes” has been often used in a less restrictive and not univocal manner with respect to the original definition centered on biogenesis (16, 35, 36). This required a great commitment of many scientists and of the International Society for Extracellular Vesicles (ISEV) itself to make order in the nomenclature and identify the minimal information for studies of EVs to avoid irreproducibility in the results (16, 36).

**Figure 1:**
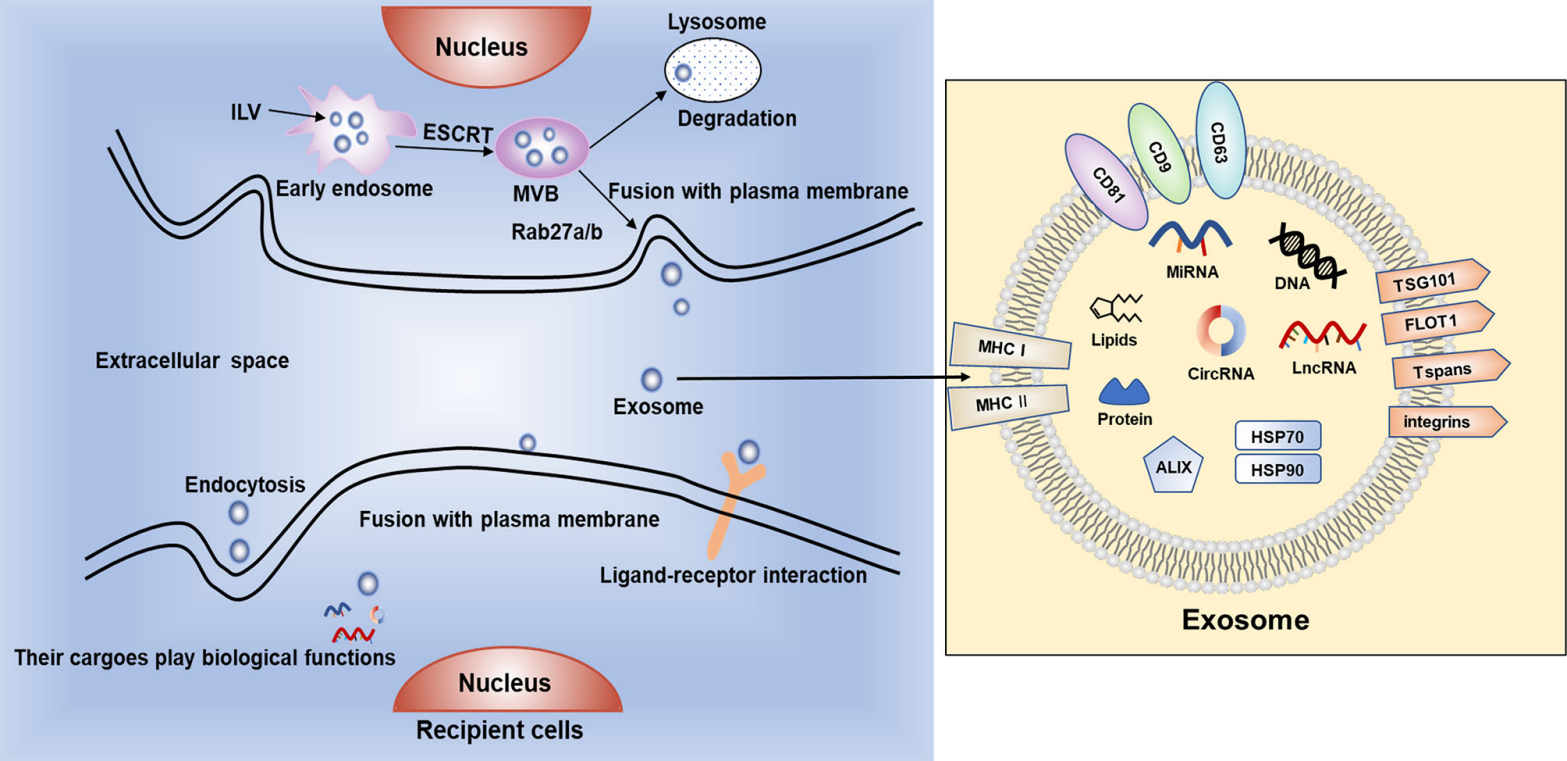
The biogenesis, secretion and uptake of exosomes. Exosomes contain bioactive cargoes including RNAs, DNAs, proteins and lipids. With the help of endosomal sorting complex required for transport (ESCRT), intraluminal vesicles (ILVs) are produced when early endosomes develop to multivesicular bodies (MVBs). Then, MVBs can be degraded by lysosomes or fuse with cell membrane and release exosomes into extracellular space. Exosomes can be ingested by recipient cells by endocytosis or fusing with plasma membrane, or through ligand–receptor interaction to exert various biological functions.

Exosomes are endosome-derived and originate inside multivesicular bodies (MVBs) secreted in physiological as well as pathological conditions (37). Exosomes (**Figure 1**) are in the form of intraluminal vesicles (ILVs) when early endosomes get maturation into late endosomes by specific signals stimulation (14). Their secretory process contains a series of procedures, start with the development of endocytic vesicles by inward budding of MVBs, then late endosomes moving along microtubules, fusing with the plasma membrane and at last releasing to the extracellular space (38, 39). In recent years, it has been increasingly confirmed that exosomes played a central part in modulating the crosstalk between tumor cells and stromal cells in the tumor microenvironment (TME) and participating in modulating various of biological processes related to cancer, such as cell proliferation, angiogenesis, metastasis and drug resistance (40, 41).

Exosomes are originally endocytic and usually contain proteins associated with endosomes including ALG-2 interacting protein X (ALIX), major histocompatibility complex class I and class II molecules (MHC I/II), Rab GTPases, tumor susceptibility gene 101 protein (TSG101), Annexins, flotillin (FLOT1), integrins and tetraspanins (Tspans) such as CD9, CD63, CD81 (42). The mechanism of exosomal biogenesis is mostly mediated by endosomal sorting complex required for transport (ESCRT). The ESCRT system mainly comprises of 4 core components, named ESCRT 0, I, II and III respectively. ESCRT-0 and ESCRT-I take charge of clustering cargoes and recruiting ubiquitylated transmembrane cargoes. ESCRT-II participates in initiating the process of inward budding, and ESCRT-III is thought to be associated with vesicle scission and membrane budding (43–45). These multiple protein complexes work synergistically with VTA1, VPS4 and ALIX to help protein ubiquitination and classify the proteins as ILVs (46). Additionally, Ca2+ can be used to regulate the fusion process of secretory lysosomes with plasma membrane and increased level of intracellular Ca2+ can induce the secretion of exosomes (47, 48).

Once released in the extracellular environment, exosomes can interact with recipient cells by recognition and conjugation to membrane-bound receptors, thus activating specific signal pathways in target cells. Some of the widely discussed mechanisms for exosome uptake are phagocytosis, macropinocytosis, clathrin-mediated endocytosis (CME), caveolin-dependent endocytosis (CDE), and plasma membrane fusion. The exact mechanism of exosome uptake by recipient cells is yet to be elucidated (49). The cargoes can induce a series of phenotypic changes in recipient cells (50). It has been reported that tumor cell-derived exosomes (TDEs) can alter the behavior of peripheral stromal cells, eventually creating an appropriate microenvironment for tumor growth (51). TDEs also involve in eukaryotic intercellular crosstalk, tumor angiogenesis, treatment resistance and tumor metastasis (52–54). Studies have shown that high levels of exosomes can be released by tumor cells and the ingredients of exosomes change under different pathological and physiological conditions (55, 56).

To data, several conventional techniques have been employed to separate exosomes, including ultracentrifugation-based separation, immunological separation, ultrafiltration separation, size-exclusion chromatography (SEC), and polymer-based precipitation separation (57). Among these techniques, the ultracentrifugation-based separation technique is the most commonly used technique for separating exosomes. However, the ultracentrifugation-based separation technique is not suitable for clinical diagnosis because of the time consuming (>4h), low recovery rate (5–25%) and poor repeatability. With the rapid development of microfluidics, microfluidics-based techniques have received increasing attention. Microfluidics-based techniques for exosome separation rely on the physical and chemical features of exosomes, such as size, density, and surface antigens present (58). However, it is difficult to completely isolate exosomes from blood samples because there are many biomolecules or biological nanoparticlesin plasma/serum that have similar size and/or density to EVs, including lipoproteins, subcellular organelles, cellular debris and viral particles (59). Among them, low/very low/high density lipoproteins (LDL, VLDL, HDL) represent ones of the most common contaminants in EV isolation as there are∼10^16^ lipoproteins/ml in plasma (60). The separation of exosomes from serum requires successful separation of exosomes from lipoproteins. By optimizing the pore size, increasing bed volume and bulk elution volume of column resin, Cui and coworkers improved SEC and developed dichotomic SEC. This method could isolate exosomes from serum with high purity and particle recovery rate, which is expected to accelerate the clinical research and achievement transformation of EVs (61).

Simultaneously, the conventional detection techniques for analyzing and quantifying exosomes include scanning electron microscopy (SEM), transmission electron microscopy (TEM), atomic force microscopy (AFM), enzyme-linked immunosorbent assay (ELISA), dynamic light scattering (DLS), and nanoparticle tracking analysis (NTA). On the other hand, microfluidics-based exosome detection methods have the advantages of high-throughput capacity, high sensitivity, low reagent consumption and portability, which include fluorescence imaging, colorimetric detection, optical special effects methods, magnetic detection and electro-chemical detection. Microfluidics combined with conventional exosome separation techniques are expected to be a research hotspot in separation and detection of exosomes (58).

Exosomal ncRNAs could be extracted from exosomes by RNA extracting kits or Trizol after separating exosomes and quantified by next-generation sequencing (NGS) (62), microarray (63), real-time quantitative polymerase chain reaction (qPCR) and digital PCR (64).

## MiRNA

**Figure 2:**
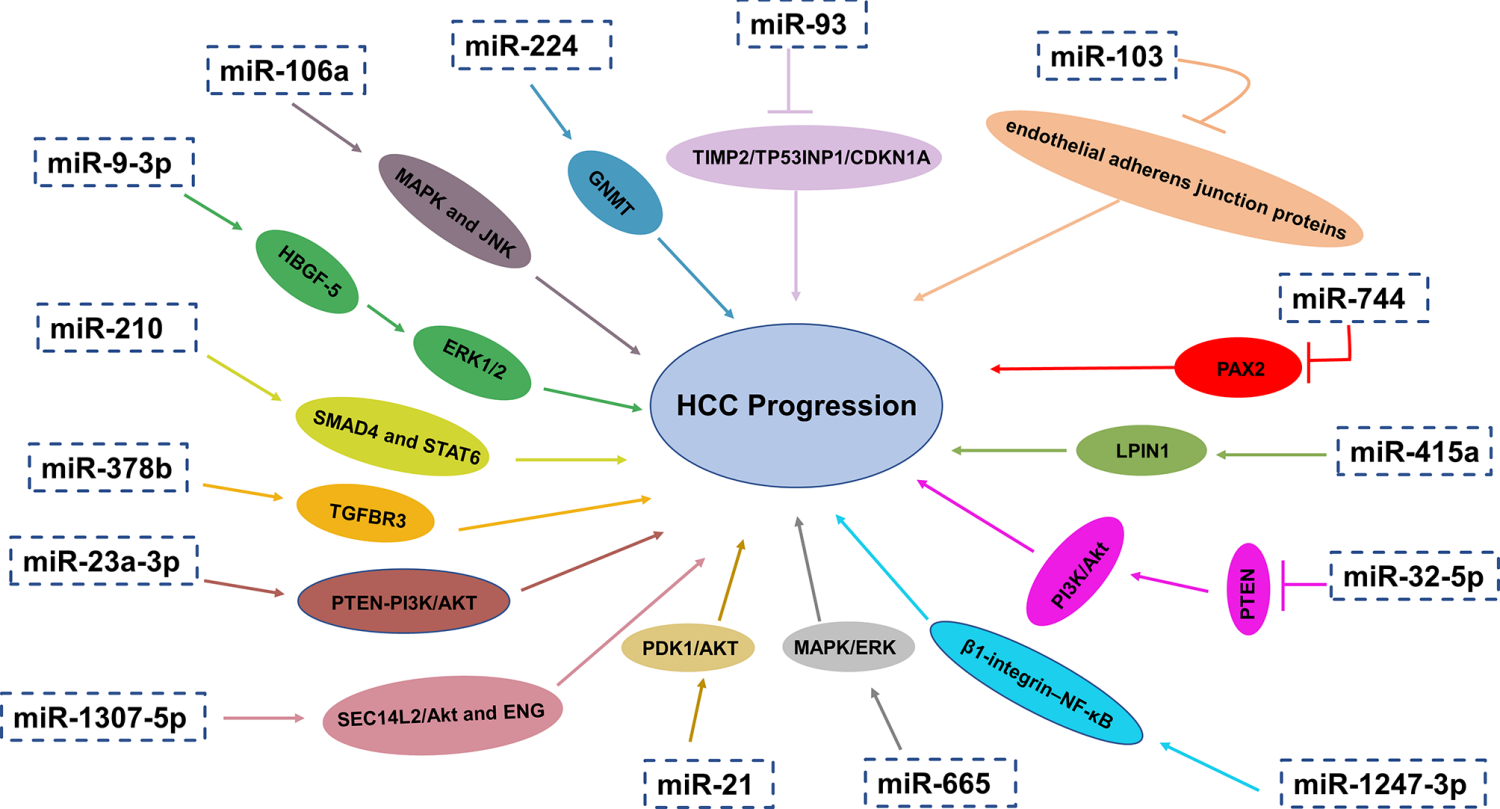
Exosomal miRNAs play a crucial role in HCC progression through different pathways and mechanisms.

The first discovery of miRNA was reported by Lee and coauthors in 1993 that small RNA molecules were encoded by gene lin-4 to regulate protein lin-14 expression in Caenorhabditis elegans (65). MiRNAs are small single-stranded RNA molecules without the ability of encoding proteins. They usually have a length of approximately 20–24 nucleotides, which modulate gene expression post-transcriptionally by complementary binding to 3′untranslated regions (UTR) of target mRNA’s and guide degradation of target mRNAs (66–68). MiRNAs may have carcinogenic effects or play the role of tumor suppressors under different conditions (69). Alteration in miRNA expression levels have been recognized as critical factors for many aspects of tumorigenesis and development such as tumor angiogenesis, immune disturbance, metastasis and so on (66–68). MiRNAs in exosomes have been considered as critical biologically active content of exosomes and this has become a hotspot in recent years. Research in recent years has showed that miRNAs in exosomes can influence a lot of aspects of liver cancer physiologically and pathologically. What’s more, exosomal miRNAs can not only reflect aberrant intrahepatic regulation but also can affect other organs and their microenvironment. More importantly, previous studies have revealed that serum miRNAs are enriched in the exosomes and can be protected from degradation by RNase in circulation (70). Taken together, exosomal miRNAs have the potential diagnostic and therapeutic value (**Figure 2**).

### MiRNAs Regulate Angiogenesis

Exosomal miR-210 was upregulated in HCC patients and related to microvessel density (MVD). Exosomal miR-210 could promote proliferation, migration and tube formation of endothelial cells in HCC cells *via* targeting SMAD4 and STAT6 in endothelial cells, which are critical for angiogenesis (71). In addition, high levels of exosomal miR-378b facilitates the angiogenesis and progression of HCC cells, which may target and negatively associated with TGFBR3, offering therapeutic strategies for HCC treatment (72). On the contrary, in the serum exosomes of patients with liver cancer, the expression of miR-451a was remarkably reduced and low level of miR-451a was associated with later clinical stages and high T classification. As a tumor suppressor, exosomal miR-451a targets LPIN1 to suppress hepatocellular tumorigenesis by regulating tumor cell apoptosis and angiogenesis (73).

### MiRNAs Mediate Drug Resistance

Recent study by Wang and coauthors demonstrated that the level of exosomal miR-744 in liver cancer tissues and serum related to the propagation and the sorafenib resistance of HCC cells was also remarkably decreased. They also reported that miR-744 directly targeted PAX2. Compared with adjacent normal tissues, PAX2 in HCC tissues increased significantly. Intriguingly, the proliferation and drug resistance of liver cancer cells were dramatically inhibited when treated with miR-744-enriched exosomes (74).

Fu and coauthors have discovered that exosomal miR-32-5p was significantly overexpressed in HCC and neighboring tissues while PTEN was significantly lower-expressed. Furthermore, upregulated expression of miR-32-5p and downregulated expression of PTEN had a positively correlation to unfavorable prognosis of patients with HCC. They also found that miR-32-5p, targeting PTEN, caused multiple drug resistance through the activation of PI3K/Akt pathway and going through epithelial-mesenchymal transition and angiogenesis. However, how miR-32-5p converts sensitive cells to resistant cells is still confusing (75). In another study, exosomal miRNA-21 could convert normal hepatic stellate cells (HSCs) into cancer-associated fibroblasts (CAFs) *via* directly targeting PTEN, resulting in activating PDK1/AKT signaling pathway in HCC. In addition, by secreting angiogenic cytokines such as VEGF, TGF-β, bFGF, MMP2 and MMP9, activated CAFs further promoted cancer progression. Furthermore, high expression of exosomal miRNA-21 was linked with low survival rate, which has guiding significance for effective prevention and treatment strategies (76).

### miRNAs and HCC Metastasis

Epithelial-mesenchymal transition (EMT) is a conservative process in which epithelial cells transition to a mesenchymal cell state, resulting in decreased intercellular adhesion and increased motility. Accumulative evidence indicates that inappropriate activation of EMT is associated with malignant transformation and the initiation of metastasis (77). It has been demonstrated that TDEs carry a pro-EMT programme including hypoxia-inducible factor 1 alpha (HIF1a), transforming growth factor beta (TGFb), caveolin-1 and b-catenin,which enhances the ability of recipient cells to invade and migrate and facilitate matrix remodelling and the formation of premetastatic niche (78).

Pulmonary metastasis is the most common remote invasive progression and one of the leading reasons of deaths related to HCC (78, 79). The study from Fang T confirmed that through reducing the expression of its target B4GALT3, the miR-1247-3p derived from tumor exosomes transform fibroblasts to CAFs. Consequently, CAFs in the lung metastatic niche are activated by activating β1-integrin–NF-κB signaling pathway. Additionally, CAFs promote tumorigenicity, chemoresistance, stemness and EMT of tumor cells by increasing secretion of IL-6 and IL-8. What’s more, upregulation of exosomal miR-1247-3p is positively related with lung metastasis in liver cancer patients (80). Also, according to the research proposed by Fang and coworkers, the level of exosomal miR-103 in HCC cells was upregulated. Exosomal miR-103 could be delivered into endothelial cells to reduce the integrity of endothelial junction and increase the permeability of vessels by targeting zonula occludens-1 (ZO-1), VE-Cadherin and p120-catenin. As a result, the transendothelial infiltration of HCC cells increased and metastasis occurred (81). In addition to the aforementioned, exosomal miR-1307-5p was reported to promote metastasis and help predict metastasis in HCC, which was dramatically overexpressed in the metastasis group (p = 0.04), as well as in the vascular invasion and tumor recurrence groups. SEC14L2/Akt and ENG-related signaling pathways were identified as downstream pathways of miR-1307-5p for promoting the EMT in HCC by comprehensive bioinformatics analysis (82).

### miRNAs Modulate Tumor Immunity

To the best of our knowledge, the effective treatment of liver cancer is living donor liver transplantation (LDLT), facing the problem of recurrence after transplantation. According to Nakano and coworkers, elevated levels of AFP (>24.1ng/ml) and exosomal miR-92b (>23.4-fold change) in HCC patients before LDLT is one of risk factors for early recurrence of HCC after transplantation and the accuracy of prediction was great with the area under the curve (AUC) values reaching 0.760. It is demonstrated by ROC curve (receiver operating characteristic curve) that there is a great predictive accuracy of posttransplant miR-92b (>41.8-fold change) for early recurrence of HCC after transplantation (AUC= 0.925). But exosomal miR-92b combined with AFP are not enough to predict the recurrence of advanced HCC after transplantation. MiR-92b in exosomes may have effects on the activity of NK cells by inhibiting CD69 expression and NK cell-mediated cytotoxicity, which is crucial for tumor immune surveillance. Therefore, exosomal miR-92b may function as prospective biomarkers for early predicting HCC recurrence after transplantation (83). Under Endoplasmic reticulum stress, miR-23a-3p derived from exosomes in HCC could increase PD-L1 expression in macrophages through PTEN-PI3K/AKT pathway, thereby suppressing T cell function (84).

### miRNAs as Diagnostic and Prognostic Biomarkers for HCC

MiR-93 in the exosomes of HCC patients was overexpressed, which was associated with the clinical characteristics of HCC such as the size and stage of tumor stage as well as survival rate. By targeting TIMP2/TP53INP1/CDKN1A, exosomal miR-93 may increase the ability of proliferation and invasion of liver cancer. It is also revealed that the expression level of exosomal miR-93 is a reliable biomarker for HCC patients related to tumor size and advanced TNM stage. Liver cancer patients with higher exosomal miR-93 have poorer prognosis. In addition, they also found that the 2-year overall survival rate of liver cancer patients who had higher serum exosome miR-93 expression was lower than that of HCC patients with lower expression of serum exosome miR-93 (85). One previous study investigated the levels of miR-665 in the serum exosomes of HCC patients and the possibility as a promising diagnostic biomarker and prognostic factors for HCC was assessed. Researchers demonstrated that the level of miR-665 in serum exosomes of HCC group was obviously higher than that of normal control group (NC), which can stimulate HCC cells proliferation and the growth of tumors through MAPK/ERK pathway. The overexpression of miR-665 in exosomes had a positive relation with local invasion, tumor size and clinical stage and had an inverse relation with survival time (86).

Cui and coauthors found that compared to the normal hepatic cell line, the level of exosomal miR-224 was remarkably elevated in HCC lines, especially in HCC patients who have larger tumors and later stages. HCC patients with increased levels of miR-224 in the exosomes had low overall survival time according to Kaplan-Meier survival curves. The feasibility of miR-224 in exosomes to differentiating HCC patients from healthy controls was evaluated with an AUC of 0.910. Exosomal miR-224 could directly target glycine N-methyltransferase (GNMT), increasing the proliferation and invasion of liver cancer cells. Thus, miR-224 in the serum exosomes can serve as a potential diagnostic biomarker and an independent prognostic factor for HCC (87). However, its capability of differentiating liver diseases needs further evaluation. Additionally, the level of exosomal miR-148a and miR-1246 were remarkably higher from liver cancer patients than that of normal individuals and patients with liver cirrhosis. Exosomal miR-148a could discriminate HCC from LC (AUC=0.891), significantly higher than AFP (AUC=0.712), compared with miR-1246 (AUC=0.785). AFP combined with miR-122 and miR-148a was the greatest diagnostic group (AUC=0.947). The sensitivity and specificity of it were 87.0% and 90.0% respectively (88). Tang and coauthors have discovered that compared with normal donors, the level of miR-9-3p in the serum exosomes of liver cancer patients was significantly downregulated (p<0.01). Exosomal miR-9-3p targets the expression of fibroblast growth factor 5 (FGF5) to suppress HCC cell proliferation and the activation of the extracellular signal-regulated protein kinase1 and 2(ERK1/2) signaling pathway. The diagnostic ability of exosomal miR-9-3p was further evaluated with 67.31% sensitivity and 61.21% specificity (89).

Liu and coworkers have reported that the level of exosomal miR-125b in patients with HCC is remarkably lower than that in patients with chronic hepatitis B (CHB) and LC. Additionally, the low expression of serum exosomal miR-125b is closely related to tumor differentiation, tumor number and TNM stage. The Kaplan-Meier curve of time to relapse (TTR) and overall survival (OS) shows that the expression level of exosomal miR-125b was positively related with HCC patient’s TTR and OS, which can independently predict TTR and OS in patients with liver cancer (90). Furthermore, it is reported by Min and coauthors that levels of serum exosomal miR-638 from patients with HCC were remarkably lower than that of healthy controls, which predicts poor prognosis in HCC. Therefore, serum exosomal miR-638 was an innovative cancer biomarker and an independent prognostic factor for HCC patients. Nevertheless, the underlying molecular mechanisms and potential targeted genes related to miR-638 in HCC continue to be elusive to date and need to be further investigated (91).

Moreover, the levels of exosomal miR-106a, miR-122, miR-125b, miR-145, miR-192, miR-194, miR-17-5p and miR-29a were dramatically increased in liver cancer patients. What’s more, these exosomal miRNAs apart from miR-145 could distinguishing HCC from healthy subjects with an AUC of 0.650 to 0.850. What’s more, it was reported that the elevated levels of exosomal miR-106a, miR-17-5p and miR-194 were associated with larger tumor size (>3 cm). In further, exosomal miR-106a facilitates tumorigenesis *via* modulating MAPK and JNK pathways, serving as a promising serum prognostic biomarker and providing a novel insight into the therapy of HCC (92). Further effort is required to validate the specificity and sensitivity of these biomarkers in prospective studies with larger patient cohorts and standardized methodology. Currently, several miRNA-based therapies for different cancers have entered into clinical trials.

## LncRNA

Long non-coding RNA (lncRNA) is a non-protein transcript longer than 200 nucleotides in length (93). LncRNAs can be found in the nucleus and cytoplasm, and exert different functions according to their subcellular localizations. lncRNAs may be involved in transcriptional regulation of gene expression and mRNA splicing in the nucleus. While in the cytoplasm, they could influence mRNA stability and regulate protein function (94). LncRNAs play its regulatory role mainly through multiple molecular mechanisms including binding with DNA to modulate gene transcription, acting as the competing endogenous RNA (ceRNA) or miRNA sponges to regulate gene expression at posttranscriptional level, associating with proteins, and encoding functional small peptides (95). LncRNAs are involved in various biological processes, such as cell proliferation, apoptosis, metastasis and regulate the tumor microenvironment in HCC, eventually causing tumor development (96). Exosomal lncRNAs obtained by the recipient cell will play its cancer-related role in the recipient cell to regulate cancer progression (**Table 1**).

**Table 1:** Biological functions and clinical applications of exosomal LncRNAs in liver cancer.

Name	Dysregulation	Significance	Reference
lnc-FAM72D-3	↓	promote cell proliferation	(97)
lnc-EPC1-4	↑	inhibit cell proliferation	(97)
lncRNA TUC339	↑	promote cell proliferation and inhibit immune response	(98, 99)
lncRNA H19	↓	promote HCC progression	(100)
linc-FAM138B	↓	inhibit HCC progression	(101)
lncRNA FAL1	↑	increase HCC cell proliferation and migration	(102)
Linc-VLDLR	↑	chemotherapeutic drugs resistance	(103)
linc-ROR	↑	chemotherapeutic drugs resistance	(104)
lncRNA ENSG00000258332.1	↑	diagnosis and prognosis of HCC	(105)
LINC00635	↑	diagnosis and prognosis of HCC	(105)
lncRNA HEIH	↑	diagnostic biomarker for HCV-related HCC	(106)
LncRNA 85		diagnostic biomarker for AFP-HCC	(107)
Lnc RNA SENP3-EIF4A1	↓	diagnostic biomaker	(108)
LINC00161	↑	biomarker	(109)
lncRNA-ATB	↑	prognostic biomarker	(110)
lncRNA CRNDE	↑	prognostic biomarker	(111)

The symbols ↑ means “upregulate” and ↓ means “downregulate”.

### LncRNAs Influence Proliferation and Migration of HCC Cells

Lnc-EPC1-4 overexpression inhibits cell proliferation and stimulates apoptosis, suggesting its functional role as a tumor suppressor gene. In contrast, knockdown of lnc-FAM72D-3 can reduce cell viability and promote cell apoptosis, indicating that lnc-FAM72D-3 acts as an oncogene in HCC (97). It has been proven previously that exosomal lncRNA TUC339 is rich in HCC patients and can promote cell proliferation and decrease cell adhesion to the extracellular matrix (ECM), which leads to the spread of HCC (98). Macrophages are imperative in the innate immune defense and have received increasing attention in the tumor microenvironment. In response to tumor-derived stimuli, macrophages can be polarized into the classical (M1) or alternative (M2) phenotypes. M1 macrophages exhibit anti-tumor activity, whereas M2 macrophages can promote tumorgenesis and tumor progression (112). Recently, Li and coauthors found that exosomal lncRNA TUC339 can be transferred to neighboring macrophages where it regulates the M1/M2 polarization and inhibits the anti-tumor immune response *in vitro* (99). It has also been shown that exosomal lncRNA H19 promoted the development of HCC including proliferation, invasion and migration and suppressed HCC cells apoptosis treated with propofol through upregulating LIM domain kinase 1 (LIMK1) *via* sponging miR-520a-3p (100). In another study, exosomal linc-FAM138B were reduced and inhibited the proliferation, migration and invasion of HCC cells *via* regulating miR-765 (101). On the contrary, exosomal lncRNA FAL1 was elevated in patients with HCC, which functioned as an oncogene and promoted the proliferation and migration of HCC cells through binding to miR-1236 competitively, and subsequently increased the expression of its target gene AFP and ZEB1 (102).

### LncRNAs Induce Chemotherapeutic Drugs Resistance

Other groups reported that exosomal linc-VLDLR could induce resistance to chemotherapeutic drugs (camptothecin and doxorubicin) and sorafenib targeted therapy in HCC. Exosomal linc-VLDLR derived from HCC cell lines were overexpressed when exposed to camptothecin, doxorubicin and sorafenib (103). The same research team also discovered another lncRNA, linc-ROR that contributes to doxorubicin and sorafenib resistance in HCC. The underlying mechanism is that TGF-β could enrich linc-ROR in exosomes from HCC, leading to an increase in the number of CD133+ liver cancer stem cells and reduced chemotherapy-induced cell death through inhibiting p53 (104). Although exosomal lncRNAs haven’t been investigated exhaustively in HCC, the above-mentioned exosomal lncRNAs promote the development of exosomal lncRNAs as a biomarker, drug resistance and therapeutic target in liver cancer.

### LncRNAs Act as Biomarkers for HCC

Compared with patients with CHB, the expression of exosomal lncRNA LINC00635 and ENSG00000258332.1 of HCC patients are both higher with the AUC of 0.750 and 0.719 respectively (105). Similarly, elevated serum exosomal lncRNA HEIH levels can also distinguish HCV-related HCC patients from chronic hepatitis C (CHC) or HCV-induced cirrhosis patients (106). In the recent studies, Huang and coauthors reported that lncRNA 85 promotes proliferation, apoptosis and metastasis of HCC cells by modulating the expression of target genes associated with miR-324-5p. LncRNA 85 was highly expressed in both AFP+ and AFP- HCC-exosomes. The specificity and sensitivity of lncRNA 85 in diagnosing AFP+ HCC were 76.7% and 80.5% respectively (cut-off = 1.650) while specificity and sensitivity were 76.7% and 80.0% respectively (cut-off = 1.645) in AFP− HCC. All these findings indicated that lncRNA 85 is capable of serving as a promising biomarker in diagnosing HCC and contributing to improving the diagnostic sensitivity of AFP-HCC (107).

Exosomal lnc RNA SENP3-EIF4A1 was obviously decreased in liver cancer tissues, which can distinguish HCC patients from healthy people with the AUC reaching 0.8028. Exosomal SENP3-EIF4A1 released by normal cells was transported to liver cancer cells to induce apoptosis and attenuate the migration and invasion abilities of HCC cells and thus reverse their malignant biological behavior (P<0.05). In addition, it was able to suppress the tumor growth *in vivo* and regulate ZFP36 expression by binding with miR-9-5p competitively. To conclude, exosomal SENP3-EIF4A1 is a novel beneficial biomarker for clinical detection of HCC, and can spread from normal cells to HCC cells through exosomes, thereby *in vivo* and *in vitro* inhibiting HCC progression (108). Exosomal LINC00161 was upregulated in HCC patients compared with the controls, which may be another innovative biomarker for HCC with remarkable stability and specificity (109).

Although the treatment for HCC have been greatly improved, the prognosis of HCC is still poor. Lee and colleagues (110) provided strong evidence that exosomal lncRNA-ATB is a new independent prognostic marker and therapeutic target for HCC. The serum exosomal lncRNA CRNDE expression levels were significantly increased in patients with HCC compared with normal controls. High serum exosomal lncRNA CRNDE expression was positively associated HCC progression and poorer prognosis (111).

## CircRNA

Identified as a novel member of RNAs, circular RNAs (circRNAs) originate from pre-mRNA back splicing with considerable stability and conservation (113–116). Different from linear RNA, circRNAs have a closed loop structure without a poly A tail or 5’−3’ polarity, which are insusceptible to RNA exonuclease or RNase R-induced degradation (117). It has been revealed that exosomal circRNAs are closely related to cellular proliferation, invasion, migration, metastasis and drug resistance (118) and due to high abundance and relative stability, the combined use of exosomes and circRNAs may become the promising diagnostic and prognostic markers in cancers (119, 120).

It was first reported that circRNAs in exosomes is rich and stable on the basis of the RNA-Seq analysis from MHCC-LM3 liver cancer cells and cell-derived exosomes. By analysis of RNA-seq, we found that the ratio of circRNAs levels to linear RNA levels in exosomes was approximately 6 times higher than in producing cells. In further, the expression levels of circRNAs were almost unchanged after incubation for 24h at room temperature. These results show that exosomal circRNAs are extremely plentiful and remain stable (121). In recent years, there has been lots of research on exosomal lncRNAs and miRNAs in cancer, but relatively little attention has been paid to exosomal circRNAs. However, in recent years, the clinical application of exosomal circRNAs is in an era of rapid development (**Table 2**).

**Table 2:** List of selected exosomal CircRNAs and their role in liver cancer.

Name	Dysregulation	Significance	Reference
circRNA-100,338	↑	regulation of angiogenesis and HCC metastasis	(122)
circPTGR1	↑	HCC metastasis	(119)
circRNA 100284	↑	inhibit HCC proliferation	(123, 124)
CDR1-AS	↑	accelerate cell proliferation and migration	(125)
circ-DB	↑	promote HCC proliferation and decreases DNA damage	(126)
circ-0051443	↓	inhibit HCC progression	(127)
circ-G004213	↑	assess the efficacy of TACE	(128)
circUHRF1	↑	therapeutic strategy	(129)
circFBLIM1	↑	therapeutic strategy	(130)
circ-0006602	↑	diagnostic biomarker	(131)
circ 0070396	↑	diagnostic biomarker	(132)
circAKT3	↑	prognostic biomarker	(133)

The symbols ↑ means “upregulate” and ↓ means “downregulate”.

### CircRNAs and HCC Metastasis

CircRNA-100,338 was elevated in the exosomes from liver cancer cells with potential metastasis, which involved in regulating angiogenesis and metastasis of HCC. It was revealed that the invasive abilities of liver cancer cells can be increased or reduced by upregulation or downregulation of exosomal circRNA-100,338. RNA pull-down assay showed that circRNA-100338 can bind RNA-binding proteins including NOVA2, which was reported to regulate vascular development and lumen formation. They also found that HCC patients undergoing radical hepatectomy had continuous upregulated level of exosomal circRNA-100,338 may be at risk of poor survival and lung metastasis (122). Wang recently proved that the overexpression of exosomal circPTGR1 was linked to the clinical stage and prognosis of HCC. Furthermore, downregulation of exosomal circPTGR1 remarkably inhibited the invasion and migration of HCC cell lines. There are evidence demonstrating that the exosome circPTGR1 competed with Met to target MIR449a, causing dysregulation of the TME and promoting liver cancer metastasis (119).

### CircRNAs Involved in Cell Proliferation and Apoptosis in HCC

Several studies in recent years have proven that circRNAs can absorb miRNAs by stable complementary binding and sponge miRNAs to modulate gene expression (134, 135). For example, exosomal circRNAs derived from adipocytes play a momentous role in promoting the tumor growth of liver cancer *via* absorbing miR-34a and activating the USP7/Cyclin A2 pathway (126). What’s more, Dai found that exosomal circRNA 100284 acted as a sponge for miR-217 to regulate cell cycle and inhibited cell proliferation *via* inducing the G2/M phase of cell cycle arrest and targeting EZH2 in HCC (123, 124). Exosomal CDR1-AS could promote HCC progression. In HCC cells, upregulated CDR1-AS accelerated cell proliferation and migration by sponging of miR-1270, increasing the expression of alpha-fetoprotein (125). It is worth noting that not only exosomal circRNAs derived from tumors, but also exosomal circRNAs derived from adipocytes, promote tumor growth. For example, the expression level of exosomal circ-deubiquitination (Circ-DB) was high in HCC patients with higher body fat ratios. Circ-DB derived from adipocytes promoted HCC proliferation and reduces DNA damage *via* functioning as a spong of miR-34a, thereby activating the USP7/Cyclin A2 signaling pathway (126). Furthermore, circ0051443, secreted by normal hepatocytes and transferred to adjacent HCC cells, was decreased in plasma exosomes of HCC patients, causing tumor cell apoptosis and cell cycle arrest through sponging of miR331-3p and upregulation of BAK1 expression (127). However, the specific functions of exosome circRNAs are under intense investigation.

### CircRNAs and Treatment

As the largest immune organ in human body, liver plays a protective role by promoting immunotolerance under physiologic conditions (136). More recently, immunotherapy has emerged as a promising therapeutic option for advanced HCC patients. Several novel immunotherapeutic methods, including the use of immune checkpoint inhibitors, new types of immune cell adoption, such as chimeric antigen receptor T cell (CAR-T), TCR gene-modified T cells and stem cells, and microRNAs have been used in clinical trials for the treatment of HCC (137). Zhang and colleagues found that circUHRF1 was overexpressed in HCC tissues and HCC-derived exosomes and inhibited NK cell function by upregulating the expression of TIM-3 *via* degradation of miR-449c-5p. Furthermore, exosomal circUHRF1 may drive resistance to anti-PD1 immunotherapy, providing a potential therapeutic strategy for patients with HCC (129).

TACE is the treatment of choice for intermediate-stage HCC, including unresectable multinodular HCC without extrahepatic spread. However, it is difficult and subjective to decide whether to repeat or stop TACE (138). It has been proved that circ-G004213 in exosomes could promote cisplatin sensitivity *via* regulation of miR-513b-5p/PRPR39 and was positively associated with the prognosis of HCC patients following TACE. Exosomal circ-G004213 may be an indicator for predicting the efficacy of TACE in patients with HCC (128).

Glycolysis is a hallmark feature of cancer especially cancer cells preferentially use glycolysis to produce glucose-dependent ATP (139). Lactate is an important metabolite in cancer metabolic reprogramming, and its large accumulation causes extracellular pH in the TME being acidified, ranging from 6.0 to 6.5 (140). Besides, TDEs are strongly connected with the TME. For example, exosomal circFBLIM1 was highly expressed in HCC and made contributions to the progression and glycolysis of HCC by sponging miR-338 and upregulating LRP6. The circFBLIM1/miR-338/LRP6 axis may provide a new therapeutic approach for HCC treatment (130).

### CircRNAs Serve as Promising Biomarkers

Exosomal circ-0006602 was generally upregulated in HCC and combined with AFP can greatly improve the accuracy of early diagnosis of HCC, having the potential to become non-invasive biomarker for the early diagnosis and screening of liver cancer (131). Similarly, exosomal circ 0070396 expressed dramatically higher in HCC samples compared with corresponding controls and displayed a better diagnostic performance than AFP and their combination provided higher diagnostic value. Moreover, the exosomal circ 0070396 expression was positively correlated with HCC progression (132).

Additionally, the levels of circulating exosomal circAKT3 in patients with HCC were significantly higher than in healthy subjects. High expression of circulating exosomal circAKT3 is associated with a higher risk of HCC recurrence and mortality. Follow-up of patients with HCC have high circAKT3 expression after surgery may help to reduce the risk of recurrence and improve prognosis (133).

## Conclusions and Perspectives

Extensive evidence has revealed ncRNAs in exosomes function as diagnostic and prognostic markers in different malignancies, including lung cancer, gastric cancer, breast cancer, liver cancer, colorectal cancer and so on. In recent years, tumor-associated cargoes in exosomes, especially with regard to ncRNAs have been a research hotspot. In this review, we talk about the clinical applications of exosomal ncRNAs in liver cancer.

Exosomes are nanometer-sized membrane vesicle structure secreted from most cell types which regulate the activation of corresponding signaling pathways in recipient (target) cells. Exosomes serve as mediators in intercellular communications both locally and systemically in the tumor microenvironment by transferring information cargo, such as proteins, lipids and diverse forms of RNAs, contributing to the progression of HCC. Because ncRNAs are stable in serum exosomes and exhibit distinct expression profiles that reflect the characteristics of cancer cells, they could function as noninvasive and sensitive ideal biomarkers for the early diagnosis and prognosis of liver cancer. The study of exosomes is still in its infancy. With the further development of the research, its role in the molecular pathogenesis of liver cancer will be clarified. Moreover, exosomes can not only be used as vaccines or drug carriers, but also are expected to transfer target genes into exosomes through genetic engineering technology for gene therapy of liver cancer. Given the above, exosomal ncRNAs provide emerging diagnostic methods and treatments for HCC.

As we further understand the nature of exosomes, diagnostic and therapeutic techniques related to exosomes are being constantly improved. Increasing emphasis has been given to exosomal microRNAs and many exosomal microRNAs have been explored in the recent years. As noninvasive, specific and sensitive tumor markers, exosomal lncRNAs have attracted more and more attention. Liquid biopsy of serum or plasma specimens is usually used for exosomal lncRNAs detection, which has the advantages of non-invasive, repeatable detection and real-time monitoring. Combined with imaging data and traditional tumor marker detection, it is expected to realize early diagnosis, recurrence monitoring, prognosis judgment and efficacy evaluation of tumors. Exosomal lncRNAs and circRNAs have a great research prospect in cancers, but a lot of lncRNAs and circRNAs have not been explored in liver cancer. At present, there are relatively few studies on the association between exosomal ncRNAs and immunotherapy in HCC. The following research can focus on this aspect to open a new chapter in liver cancer immunotherapy. In addition, there is a lack of standard methods for exosome isolation and purification. To solve the present problem, first of all, it is necessary to identify, isolate and quantify exosomes accurately, efficiently and selectively. What’s more, the detection technology of lncRNAs and the specificity of exosomal ncRNAs as biomarker of HCC needs to be improved. Both human tumor cells and normal cells can secrete exosomes, but there is no specific marker to distinguish the source of exosomes at present. Therefore, how to avoid the interference caused by exosomes secreted by normal cells and separate tumor-specific exosomes is also a major challenge. Many experiments are done in mice or *in vitro* and the application of exosomal lncRNA in the treatment of liver cancer needs to be further verified *in vivo*.

## Author Contributions

RW conceptualized the review. WW was the major contributors in writing the manuscript. WW designed the figures. L-PH prepared the tables. HS and X-YC critically reviewed and edited the manuscript. All the authors read and approved the final manuscript.

## Funding

This study was supported by grants from the National Natural Science Foundation of China (Nos. 81772995 and 81472266); the Excellent Youth Foundation of Jiangsu Province, China (No. BK20140032); Jiangsu Province’s Key Provincial Talents Program (No. ZDRCA2016090).

## Conflict of Interest

The authors declare that the research was conducted in the absence of any commercial or financial relationships that could be construed as a potential conflict of interest.

## Publisher’s Note

All claims expressed in this article are solely those of the authors and do not necessarily represent those of their affiliated organizations, or those of the publisher, the editors and the reviewers. Any product that may be evaluated in this article, or claim that may be made by its manufacturer, is not guaranteed or endorsed by the publisher.
